# Radiotherapy and the cellular DNA damage response: current and future perspectives on head and neck cancer treatment

**DOI:** 10.20517/cdr.2020.49

**Published:** 2020-09-17

**Authors:** Maria Rita Fabbrizi, Jason L. Parsons

**Affiliations:** ^1^Cancer Research Centre, Department of Molecular and Clinical Cancer Medicine, University of Liverpool, Liverpool L3 9TA, United Kingdom.; ^2^Clatterbridge Cancer Centre NHS Foundation Trust, Clatterbridge Road, Bebington CH63 4JY, United Kingdom.

**Keywords:** DNA damage, DNA repair, head and neck cancer, ionising radiation, proton beam therapy, radiobiology, radiotherapy

## Abstract

Incidences of head and neck squamous cell carcinoma (HNSCC) have been on the rise in the last few decades, with a significant risk factor being human papillomavirus (HPV) type-16/18 infection, particularly in the development of oropharyngeal cancers. Radiotherapy (RT) is an important treatment modality for HNSCC, where it promotes extensive cellular DNA damage leading to the therapeutic effect. It has been well-established that HPV-positive HNSCC display better response rates and improved survival following RT compared to HPV-negative HNSCC. The differential radiosensitivity has been largely associated with altered cellular DNA damage response mechanisms in HPV-positive HNSCC, and particularly with the signaling and repair of DNA double strand breaks. However, other factors, particularly hypoxia present within the solid cancer, have a major impact on relative radioresistance. Consequently, recent approaches aimed at enhancing the radiosensitivity of HNSCC have largely centered on targeting key proteins involved in DNA repair, DNA damage checkpoint activation, and hypoxia signaling. These studies have utilised *in vitro* and *in vivo* models of HPV-positive and HPV-negative HNSCC and examined the impact of specific inhibitors against the targets in combination with radiation in suppressing HNSCC cell growth and survival. Here, accumulating evidence has shown that targeting enzymes including poly (ADP-ribose) polymerase, ataxia telangiectasia and Rad-3 related, DNA-dependent protein kinase catalytic subunit, and checkpoint kinase 1 can radiosensitise HNSCC cells which should be taken forward in further preclinical studies, with the goal of optimizing the future effective RT treatment of HNSCC.

## Introduction

Head and neck cancer describes several different malignant tumors that develop in or around the throat, larynx, nose, sinuses, and mouth. They usually develop in the squamous cells of the mucosal surfaces and therefore are often referred to as head and neck squamous cell carcinoma (HNSCC). In the UK, HNSCC is the 8th most common cancer with 12,200 new cases every year, the majority occurring in men (69%). Since the early 1990s, HNSCC incidence rates have increased by ~30% in the UK. One year survival rates among HNSCC subtypes are reportedly highest in salivary gland cancer (~60%) and lowest in hypopharyngeal cancer (~20%)^[1-4]^. Tobacco and alcohol use are the common risk factors associated with HNSCC. Furthermore, infection by high-risk (type-16/18) human papillomaviruses (HPVs) causes development of HNSCC, particularly in cancers of the oropharynx (~40%-60% incidence). Importantly, patients with HPV-positive HNSCC are known to have a better prognosis and improved survival rates associated with an improved response to radiotherapy (RT) and chemotherapy as compared with HPV-negative HNSCC. This reflects the different molecular mechanisms and biological characteristics underlying the oncogenic processes^[5]^.

Treatment of HNSCC invariably include surgery, RT, and chemotherapy, either as a single treatment or in combination. The choice of one treatment over another is strongly influenced by the stage of disease at diagnosis^[6]^. While X-rays are used as a conventional RT treatment for HNSCC, major advancements have been achieved with the introduction of proton beam therapy^[7]^ and the development of 3D conformal stereotactic RT^[8]^, which allow a better and safer treatment for the patients. The introduction of adaptive RT as a special form of image-guided RT has also been significant^[9]^. Nevertheless, all RT techniques target and damage the DNA within the cancer cells leading to the therapeutic effect. Whilst RT promotes largely the formation of DNA base damage and DNA single-strand breaks (SSBs), more important is the induction of potentially toxic DNA lesions including DNA double strand breaks (DSBs) and complex DNA damage (CDD), containing multiple DNA damage types including DNA base damage, abasic sites, and SSBs and DSBs within close proximity (1-2 helical turns of the DNA). These DNA lesions trigger the activation of the cellular DNA damage response (DDR), involving several cellular proteins and pathways that attempt to repair and restore the integrity of the DNA^[10]^. Depending on which cell cycle stage the cell is in, DSBs can be resolved though the activation of two different repair pathways, namely non-homologous end-joining (NHEJ) and homologous recombination (HR)^[11-13]^. The ultimate aim of RT is to create sufficient and/or persistent DNA damage in the cancer cells that exceeds the cellular capacity for repair, which ultimately triggers the cell death response or blocks their potential to replicate. This goal can be reached more effectively by combining RT with chemotherapeutics, specifically those that inhibit the DNA repair or cell cycle machinery.

In this review, we describe the effects of RT on DNA damage and the cellular DDR pathways responsible for DNA repair. We also provide an up-to-date summary of the biological mechanisms critical in controlling radiosensitivity of HNSCC, as well as current radiosensitisation strategies being explored for improving the outcome of HNSCC patients post-RT.

## The cellular DDR following radiotherapy

RT is a non-invasive treatment method that has certain advantages over chemotherapy or surgery, especially when the cancer is not resectable, and it is the elective treatment for the majority of HNSCC. In order to kill cancer cells, RT damages the DNA either directly or indirectly through ionisation of water molecules. Low linear energy transfer (LET) radiation, including X-rays and γ-rays, deposits a relatively small amount of energy over a short distance, whereas high LET radiation, including charged particles, will deposit a large amount of energy within the same short distance^[14,15]^. Consequently, high LET radiation will display an enhanced biological effect. This is in part mediated by increases in the formation of DNA DSBs and CDD that represent a greater challenge to the cellular DNA repair machinery, whereas the majority of DNA lesions generated by low-LET radiation are DNA base lesions and SSBs that are repaired relatively more efficiently. Particle beam therapy (e.g., proton or carbon ion beams) displays another distinct advantage in that the radiation dose can be delivered directly to the cancer cells with limited exposure of the surrounding healthy tissue. This is due to the deposition of maximum energy in depth at a well-defined region, the Bragg peak, which in turn will lead to less RT-related side effects when compared to conventional RT using low-LET photons^[16]^. However, particle beam therapy exhibits increases in LET at and around the Bragg peak, which can generate a different DNA damage spectra that contributes to an increased biological response. As a consequence, there is still a significant amount of biological uncertainty when utilising this therapeutic approach^[17,18]^. Nevertheless, the main goal of RT is to either cause sufficient cellular DNA damage or enhance the persistence of the damage to greatly exceed the cells capacity for repair, thus promoting cell death.

**Figure 1:**
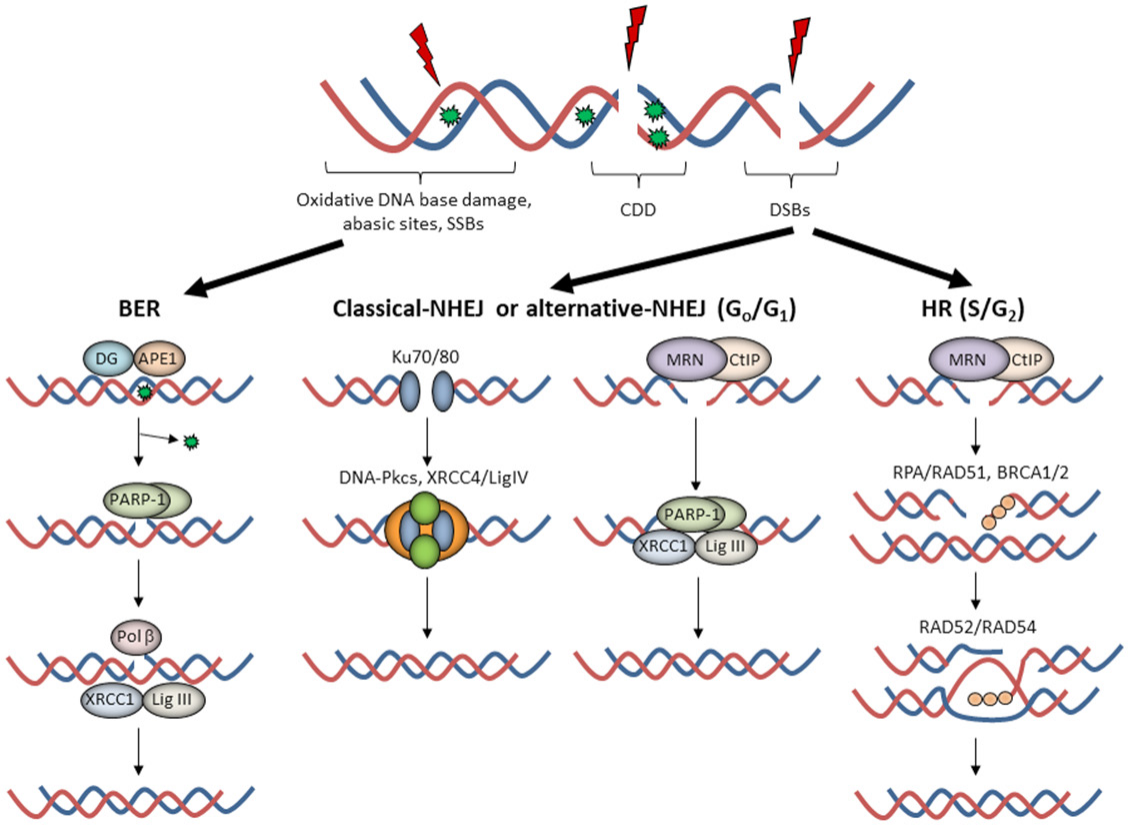
The cellular response to radiation-induced DNA damage. Ionising radiation can generate a variety of DNA lesions, but where oxidative DNA base damage, abasic sites, and single-strand breaks (SSBs) predominate. The base excision repair (BER) pathway involves recognition of the damaged base by a damage-specific DNA glycosylase (DG) and incision of the abasic site by APE1. Following SSB generation and binding by poly(ADP-ribose) polymerase-1 (PARP-1), deoxyribosephosphate (dRP) removal and gap filling is conducted by Pol β prior to ligation by XRCC1-Lig IIIα. DNA double strand breaks (DSBs) are repaired by either non-homologous end-joining (NHEJ) in G_0_/G_1_ or by homologous recombination (HR) in S/G_2_ phase of the cell cycle. In NHEJ, two general pathways exist, c-NHEJ and a-NHEJ. c-NHEJ utilises Ku70/80 to bind the DNA ends, followed by DNA-Pkcs and XRCC4-Lig IV to promote DNA ligation. On the other hand, a-NHEJ involves DSB end-resection by the MRE11/RAD50/NBS1 (MRN)-C-terminal binding protein-interacting protein (CtIP) complex, PARP-1 binding to the DSB ends, and subsequent repair by XRCC1-Lig IIIα (or Lig I). During HR, DNA end-resection by the MRN complex promotes replication protein A (RPA) and RAD51 binding to the single stranded DNA overhangs in the company of BRCA1. This promotes strand invasion into the sister chromatid through a BRCA2-dependent process and subsequent DNA synthesis in the presence of RAD52/RAD54, followed by formation and resolving of Holliday junctions, completing the DSB repair process. Of note is that complex DNA damage (CDD) can consist of several different DNA lesions in close proximity and therefore is likely to utilise both BER and NHEJ/HR for repair

The cellular DDR is an elegant signaling network able to detect and repair DNA damage, with different DNA repair pathways activated depending on the type of lesion [Figure 1]. At least 90%-95% of the DNA damage induced by low-LET RT is DNA base damage and SSBs, which are repaired through the base excision repair (BER) pathway^[19,20]^. The DNA base damage is recognized and excised by damage-specific DNA glycosylases, which along with AP endonuclease-1 (APE1), incise the phosphodiester backbone to create an SSB flanked by 3’-OH and 5’-deoxyribosephosphate (dRP) ends. Poly (ADP-ribose) polymerase-1 (PARP-1) binds to the SSB due to its high affinity for the lesion and protects the DNA ends. DNA polymerase β (Pol β) then excises the 5’-dRP ends and simultaneously inserts a new nucleotide. This is followed by X-ray cross-complementing protein 1 in complex with DNA ligase IIIα (XRCC1-Lig IIIα) which ligate the SSB ends.

In the case of DSBs, the damage is firstly recognized by the ataxia telangiectasia mutated (ATM) or ataxia telangiectasia and Rad-3 related (ATR) protein kinases that stimulate phosphorylation of the histone H2AX variant (forming γH2AX). Cells then activate either NHEJ or HR depending on whether the cell is in G_0_/G_1_ phase where NHEJ pathway is employed, or in S/G_2_ phases where a sister chromatid is available to enable HR. NHEJ can be further divided into two sub-pathways^[12]^. Canonical-NHEJ (c-NHEJ) utilises the Ku70/80 heterodimer that bind the DSB ends, followed by the DNA-dependent protein kinase catalytic subunit (DNA-Pkcs) and X-ray cross-complementing protein 4 in complex with DNA ligase IV (XRCC4/LigIV) that ligate the DSB ends. In contrast, alternative-NHEJ (a-NHEJ) is initiated by the C-terminal binding protein-interacting protein (CtIP) and MRE11/RAD50/NBS1 (MRN) complex that resect the DNA ends, followed by PARP-1 binding, gap filling by a DNA polymerase, and ultimately ligation by XRCC1-Lig IIIα (or alternatively DNA ligase I). HR is an error-free DSB repair mechanism, and similar to a-NHEJ, is initiated by the CtIP-MRN complex that promotes DNA end resection and requires breast cancer protein 1 (BRCA1). The 3’-single stranded DNA generated is then bound and stabilized by replication protein A (RPA), replaced with the recombination protein RAD51 in a BRCA2-dependent manner, which then undergoes homology search and strand invasion into the sister chromatid. DNA synthesis in the presence of RAD52/RAD54 results in branch migration and formation of Holliday junctions that are resolved by DNA resolvases that ultimately restore genome integrity. In the case of CDD, which can include a mixture of DNA lesions within a short distance on the DNA molecule, it is likely that the repair of these sites requires more than one DNA repair pathway to resolve the damage. Interestingly, we have recently demonstrated that high-LET proton irradiation generates a high frequency of CDD sites consisting of abasic sites and SSBs, which have a high dependency on PARP-1 for repair^[21]^.

## Factors contributing to HNSCC radiosensitivity

### HPV

HPV is a circular, double stranded DNA virus and a member of the papillomaviridae family. Of the over 200 types of HPV identified, HPV-16 and HPV-18 are the two that mainly contribute to oncogenesis and are the most commonly found types in HNSCC^[22]^. HPV-positive HNSCC are known to display high radiosensitivity compared to HPV-negative HNSCC as well as higher patient survival rates due to that^[23]^. Starting from these clinical observations, several investigations have been performed to unravel the molecular and biological mechanisms responsible for controlling the radiosensitivity of HNSCC, and particularly the role played by HPV in enhancing intrinsic radiosensitivity. To date, it has been largely demonstrated that patient-derived HPV-positive HNSCC cell lines are more sensitive than HPV-negative HNSCC cell lines to irradiation *in vitro*, which is consistent with the clinical observations. This suggests that these HNSCC cell lines are good models for investigating the nature of the differential radiosensitivity. As for the mechanism involved, several reports have shown that there is a defect in expression of key DSB repair effector proteins and/or defects in the efficiency of the DSB repair pathways post-irradiation. Firstly, five HPV-positive HNSCC cell lines were demonstrated to harbour more persistent levels of 53BP1 and γH2AX foci and display a marked G2/M arrest in response to radiation compared to five HPV-negative HNSCC cell lines^[24]^. This finding was replicated in another study comparing two HPV-positive with two HPV-negative HNSCC cell lines, where the HPV-positive HNSCC cell lines demonstrated delayed resolution of γH2AX and 53BP1 foci and delayed repair of radiation-induced DSBs post-irradiation revealed through the neutral comet assay^[25]^. A third study attributes the deficiency in DSB repair in two HPV-positive versus one HPV-negative HNSCC cell line to decreased BRCA2, DNA-Pkcs, and 53BP1 protein expression in HPV-positive HNSCC cells, which ultimately led to NHEJ and HR signaling deficiency as demonstrated through the lack of DNA-Pkcs and BRCA2/RAD51 foci, respectively post-irradiation^[26]^. It is interesting to note that there is evidence that HPV-positive HNSCC cells also show upregulation of several proteins involved in BER and SSB repair, including PARP-1, PNKP, Pol β, and XRCC1^[25]^. Whilst this does not appear to relate to the observed increased radiosensitivity, it suggests a compensatory mechanism of increased BER activity through loss of DSB capacity. These data are supported by analysis of the TCGA database, which demonstrated increased gene expression of at least XRCC1 and PARP-1 in HPV-positive HNSCC. Interestingly, it was further shown that shRNA-mediated downregulation of multiple BER genes enhanced the radiosensitivity of one HPV-positive HNSCC cell line and not of a corresponding HPV-negative HNSCC cell line^[27]^.

In addition to defective DSB repair pathways, differentially regulated cell cycle control in HPV-positive HNSCC cells is well established, primarily due to the impact of the E6 and E7 HPV genes. The E6 and E7 proteins can bind the tumour suppressor protein p53 and retinoblastoma (Rb) protein, respectively, facilitating their ubiquitylation-dependent proteasomal degradation resulting in the cells losing their ability to perform cell cycle arrest or to undergo apoptosis^[28]^. The oncoprotein E7 also leads to the accumulation of p16, which is used as a biomarker of HPV-positive HNSCC^[29]^. p16 overexpression in HPV-positive HNSCC cells causes initiation of cell cycle progression, but independently has been suggested to have a direct impact on DDR, specifically through HR by reducing RAD51 foci formation post-irradiation^[30]^. Interestingly, p16 has been recently demonstrated to cause downregulation in the protein levels of the E3 ubiquitin ligase TRIP12 leading to deficiencies in DSB repair. Targeting TRIP12 using siRNA in HPV-negative HNSCC cells led to an impairment of HR by inhibiting the recruitment of BRCA1 to DSBs post-irradiation^[31]^. Cumulatively, these data clearly show the impact of HPV in reducing the efficiency of the cellular DDR in HNSCC cells.

### Hypoxia

The existence of hypoxia (low oxygen) in human tumors, an important source of radioresistance, was first established in the 1950s^[32,33]^. This biological effect is caused by the physical interference of the lack of oxygen with the radiation effects on creating DNA damage, but also through the alteration of cellular pathways and processes. Oxygen is a particularly powerful radiosensitiser. However, it has been shown that HNSCC can reach levels of moderate and severe hypoxia which will ultimately lead to radioresistance and poor prognosis^[34,35]^. In terms of cellular pathways, the major player mediating the hypoxic response are the hypoxia-inducible factors (HIFs). The HIF-α subunits contain proline residues which are hydroxylated by prolyl hydroxylase domain (PHD) enzymes allowing the von Hippel-Lindau tumour suppressor protein to label it for ubiquitylation and rapid proteasomal degradation under normoxic conditions^[36-38]^. During hypoxia, the PHD enzymes are inhibited, leading to HIF-α stabilization which in turn leads to transcriptional activation of many hypoxia-related genes^[39,40]^. Interestingly, it has been observed that five HPV-positive HNSCC cell lines show higher levels of HIF1-α (but also PHD2 enzyme) compared to two HPV-negative HNSCC cells under conditions of normoxia, possibly due to the HPV oncoprotein inhibiting PHD2-dependent hydroxylation of HIF1-α. Under hypoxic (1% oxygen) conditions, relative protein levels of HIF1-α proportionately increased in both HPV-positive and HPV-negative HNSCC cell lines, with the HPV-positive cells still retaining higher levels of the protein^[41]^.

Whilst the general impact of hypoxia in mediating cellular radioresistance is well acknowledged, the molecular mechanisms affected specifically in HPV-positive in comparison to HPV-negative HNSCC cell lines, and the relationship to radiosensitivity have not been studied in detail. Hypoxia, particularly severe hypoxia (< 0.1% oxygen), promotes genetic instability through activation of ATM/ATR leading to cell cycle arrest, and downregulation of NHEJ and HR DSB repair pathways at a transcriptional and epigenetic level thus conferring a more resistant cellular phenotype^[42-46]^. Under hypoxia-induced replication stress, ATR is specifically required for phosphorylation of downstream proteins, including checkpoint kinase 1 (CHK1), p53, and γH2AX^[45,47]^. It has been demonstrated using three HPV-positive and three HPV-negative HNSCC cells, that a short term incubation (1 h) in severe hypoxia (0% oxygen) prior to irradiation, led to increased radioresistance of both sets of cell lines with observed oxygen enhancement ratios of 2.3-2.9^[48]^. This was associated with an upregulation in the expression levels of hypoxia-responsive genes, the magnitude of which varied between the cell lines used. More recently, in three HPV-negative HNSCC cell lines it was shown that hypoxia (0.1% oxygen) during irradiation increases cell survival and reduces the levels of residual γH2AX foci post-irradiation. Interestingly though, when cells were exposed to a prolonged period of 4-5 days in hypoxia (1% oxygen) prior to irradiation, ATM-deficient HNSCC cells showed increased radiosensitivity associated with a delayed phosphorylation of DNA-Pkcs and delayed NHEJ pathway activation, whilst the other two HPV-negative HNSCC cell lines displayed increased radioresistance^[49]^. This demonstrated a cell line-specific response following long term hypoxia treatment prior to irradiation. HPV-negative HNSCC usually contain a high frequency of p53 mutations, therefore the concomitant roles played by p53 mutants and HIF-1 in a hypoxia context could partially explain the higher radioresistance capacity observed in these tumors. However, more detailed cellular investigations are required.

## HNSCC radiosensitisation strategies

Enhancing the intrinsic radiosensitivity of HNSCC prior to RT by targeting key cellular pathways is an approach for combating radioresistance, with the goal of yielding more effective treatment of the tumour. Several targets have been identified and explored *in vitro*, including the epidermal growth factor receptor (EGFR), PARPs, and several proteins involved in DSB repair and cell cycle regulation (key targets and pathways summarised in Table 1). Hypoxia has also been investigated, and particularly strategies aimed at increasing the oxygen concentration in the inner cancer mass to improve RT outcome.

**Table 1:** Promising pathways and targets for radiosensitisation in HNSCC models

Pathway	Target	Compound	HPV status	Model	Ref.
Hypoxia	AKT	MK2206	HPV-positive	Cell lines	[50]
DNA repair	PARP	Veliparib	HPV-negative	Cell lines	[26,27,51]
		Veliparib	HPV-positive	Cell lines	[26,27]
		Veliparib	HPV-positive	Xenografts	[27]
		Olaparib	HPV-negative	Cell lines	[25,52,53]
		Olaparib	HPV-positive	Cell lines	[25,54]
		Niraparib	HPV-negative and HPV-positive	Cell lines	[55,56]
	ATR	VE821	HPV-negative	Cell lines	[17,57]
		VE821	HPV-positive and HPV-negative	Cell lines, 3D spheroids	[17]
		AZD6738	HPV-negative	Cell lines, 3D spheroids	[58,59]
	ATM	GSK645416A	HPV-negative	Cell lines	[60]
		KU-55933	HPV-positive and HPV-negative	Cell lines, 3D spheroids	[17]
	DNA-Pkcs	KU0060648	HPV-negative	Cell lines	[59]
		IC87361	HPV-negative	Cell lines	[61]
		NU7441	HPV-positive and HPV-negative	Cell lines, xenografts	[27]
		KU-57788	HPV-positive and HPV-negative	Cell lines, 3D spheroids	[17]
Cell cycle	CHK1	SAAR020106	HPV-negative	Cell lines, xenografts	[62]
	CHK1	CCT2444747	HPV-negative	Cell lines, xenografts	[63]
	CHK1	PF0477736	HPV-negative	Cell lines	[64,65]
	CHK1	PF0477736	HPV-positive	Cell lines	[54,65]
	CHK1	LY2603618	HPV-positive	Cell lines	[66]
	CHK1	MK8776	HPV-positive	Cell lines	[66]
	WEE1	AZD1775	HPV-positive	Cell lines	[66]
	CDK4/6	Palbocyclib	HPV-negative	Cell lines	[67]

HNSCC: head and neck squamous cell carcinoma; HPV: human papillomavirus; PARP: poly(ADP-ribose) polymerase; ATM: ataxia telangiectasia mutated; ATR: ataxia telangiectasia and Rad-3 related; DNA-Pkcs: DNA-dependent protein kinase catalytic subunit

### Epidermal growth factor receptor

EGFR is a member of the ErbB/HER family of tyrosine kinase receptors. It has been linked to cancer progression in HNSCC, and overexpression of EGFR is associated with poor prognosis and its level has been suggested to be a good predictor for patient outcome in HNSCC^[68-70]^. At a cellular level, EGFR has six known ligands, including epidermal growth factor, transforming growth factor-α, and amphiregulin. When activated EGFR affects four major signaling pathways, namely the MAPK, PI3K/AKT/mTOR, PLCγ/PKC, and the JAK/STAT pathways^[71]^. In addition, activated EGFR increases the expression of COX2 and its downstream product PGE2, which creates a positive feedback in reactivating EGFR^[71,72]^. Interestingly, nuclear EGFR has been suggested to induce proliferating cell nuclear antigen and DNA-Pkcs phosphorylation, which ultimately triggers cell proliferation and promotes the repair of DNA damage caused by chemoradiotherapy^[73]^. Therefore, the inhibition of EGFR has been suggested to be a possible therapeutic target in HNSCC. To date, two main approaches in inhibiting EGFR have been explored, namely monoclonal antibody (mAb)-based drugs and small molecule tyrosine kinase inhibitors (TKIs). mAb-based drugs act by binding the extracellular domain of EGFR and inhibiting its dimerization, which ultimately leads to its degradation after cell internalization. The TKI compounds target the intracellular tyrosine kinase domain of EGFR and compete with ATP binding to eliminate EGFR downstream signaling. The mAb cetuximab is an approved chemotherapeutic for use in combination with RT in patients with unresectable, locoregionally advanced HNSCC, as it has been proven that its administration increases survival compared to RT alone^[74]^. However, a recent study has shown that platinum-based chemoradiotherapy is superior to cetuximab treatments^[75]^. In fact, it has been observed that cetuximab caused tumor growth delay in hypopharyngeal xenografts possibly due to reduced ERK1/2 phosphorylation. However, laryngeal xenografts did not show any additive effect of cetuximab to RT and instead displayed increased proliferation, suggesting cetuximab may be effective only in a subpopulation of HNSCC^[76]^. This is further reflected by the recent failure of two phase III studies examining cetuximab in combination with RT in HPV-positive HNSCC^[77,78]^, which has raised doubts about EGFR targeting in HNSCC^[79]^. Many other mAb treatments of HNSCC have reached phase III of development and initially showed promising results^[80]^.

Several TKIs have been investigated in the last 20 years, with gefitinib and erlotinib the first to be tested, however more recent data have questioned their use in HNSCC. For example, erlotinib failed to improve the radiosensitivity of five HPV-negative HNSCC xenograft models through the observed lack of impact on tumour growth^[81]^. A recent study showed that erlotinib was unable to significantly radiosensitise fourteen HPV-negative HNSCC cell lines, although increased radiosensitisation was observed in some cells under specific re-plating conditions that overcame the G2 cell cycle arrest caused by EGFR targeting^[82]^. Additionally, the recent use of the second generation TKI afatinid in phase III trials in HNSCC gave conflicting results^[83,84]^. The observed differential responses of TKIs in radiosensitising HNSCC has therefore led to suggestions that their administration is in addition to current therapeutic treatments^[80]^. Nevertheless, results regarding both mAb and TKIs must be considered cautiously, as recent works have shown that EGFR overexpression is not common in patients with HNSCC and that in general EGFR expression and activity are not well correlated, with many cell lines used in studies not representative of the clinical situation^[85,86]^.

### Hypoxia

Hypoxia is well known to contribute to poor prognosis and treatment resistance in HNSCC, therefore hypoxic modulation has been investigated particularly in a clinical setting in order to restore sensitivity to treatment. However, oxygenation treatments are not routinely used clinically because there is no ideal technique to quantify tumor hypoxia and no effective treatment to modify hypoxic conditions. Many hypoxia targeting methodologies have been proposed and tested, including hyperbaric oxygen, hyperthermia, and agents targeting tumor blood flow^[87]^. Currently, oxygen-mimetic radiosensitisers, such as the nitroimidazoles, are the most promising compounds for overcoming hypoxia. Whilst etanidazole failed to show benefit in advanced HNSCC patients in combination with RT in a phase III clinical trial^[88]^, nimorazole has been demonstrated clinically to be an effective radiosensitiser for HPV-negative but not for HPV-positive HNSCC^[89-91]^. Following these investigations and further results of studies from the Danish Head and Neck Cancer Group, nimorazole has become standard of care to HNSCC patients in combination with RT in Denmark. A phase III trial examining nimorazole efficacy with RT in locally advanced HNSCC is currently ongoing in the UK^[92]^. Recently, a group of novel nitroimidazole alkylsulfonamides have been tested using a prodrug approach in order to increase solubility and improve drug delivery, with some of them at least showing promising results in their ability to radiosensitise anoxic colorectal cancer cells *in vitro*^[93]^.

Several other agents have been investigated for overcoming tumor hypoxia, including those targeting tumor blood flow and drugs targeting hypoxia-induced proteins. Vascular disrupting agents (VDAs) alter tumor blood flow by increasing vascular permeability through tubulin binding on endothelial cells, whilst possibly activating apoptosis^[94]^. 5,6-dimethylxanthenone-4-acetic acid (DMXAA) is a flavone acetic acid analogue that showed promising results as a monotherapy in reducing growth of HPV-negative HNSCC xenografts in mice^[95]^. DMXAA may also interact with tumour necrosis factor, leading ultimately to tumor oxygenation and increased radiosensitivity^[96]^. However, the impact of DMXAA and other VDAs in specifically radiosensitising HNSCC has not been reported. In relation to targeting hypoxia-induced proteins, the protein kinase AKT has been shown to be activated under severe hypoxia (0.1% oxygen for 6-72 h) in both HPV-positive and HPV-negative HNSCC cell lines, and that the AKT inhibitor MK2206 alone significantly reduced HPV-positive and HPV-negative HNSCC cell line growth (sensitiser enhancement ratios of ~1.5)^[50]^. Interestingly, MK2206 led to increased radiosensitivity of only the HPV-positive HNSCC cell lines under hypoxic conditions, however the study was limited to analysing the response to a single dose of radiation, and the molecular basis for this effect is currently unclear. A major problem with the clinical translation of these and other strategies is the heterogeneity of oxygen tensions within the hypoxic region of the tumour, where the level of radioresistance also varies. Nevertheless, further studies examining the molecular and biological impact of different levels of hypoxia on the comparative radiosensitivity of HPV-positive and HPV-negative HNSCC cell lines and tumours, and ultimately strategies to overcome radioresistance, are required.

## PARP-1

PARP-1 is one of the 17 members of the PARP family, which acts as an important sensor for gaps and SSBs in the DNA and plays a fundamental role in BER^[97,98]^. Here, PARP-1 uses NAD^+^ to catalyse the synthesis of ADP-ribose chains to itself and also to target residues of specific proteins, which acts as a platform for DNA repair protein recruitment. PARP1 is, however, also involved in the repair of DSBs via both HR and NHEJ^[99]^. There has been significant interest in PARP-1, and particularly PARP inhibitors, because of its synthetic lethal partnership with BRCA1/2 mutations in breast cancers^[100,101]^. Therefore, PARP inhibitors have been increasingly investigated as a treatment option in HNSCC with some promising results. Relatively earlier studies focused on veliparib which showed in three HPV-negative HNSCC cell lines that the inhibitor (at >5 µmol/L) exhibited high cytotoxicity in the presence of ionising radiation, and was exacerbated with the EGRF inhibitor cetuximab^[51]^. Similarly, veliparib (at 10 µmol/L) decreased the survival of one HPV-negative HNSCC cell line post-irradiation, but had a ~1.5-fold enhanced effect on two HPV-positive HNSCC cell lines due to their reported DSB repair defect^[26]^. This is supported by enhanced radiosensitivity of three HPV-positive HNSCC cell lines using high doses of veliparib (10 and 20 µmol/L), although one out of three HPV-negative HNSCC cell lines also displayed marked radiosensitisation^[27]^. Interestingly though, veliparib only had a relatively mild effect on suppressing growth of HPV-positive HNSCC xenografts.

Several studies have utilised the PARP inhibitor olaparib as a radiosensitizer, although these have shown mixed results particularly regarding degree of radiosensitisation of HPV-negative HNSCC cell lines, which are deemed DSB repair proficient. Indeed, it has been suggested that HPV-negative HNSCC cell lines are more responsive to olaparib (1 µmol/L) radiosensitisation when the cells are specifically HR-deficient^[52]^, although individual variability in cellular responses was observed. A recent study has shown that olaparib (at 1 and 5 µmol/L) is effective in combination with radiation in decreasing the survival of HPV-negative HNSCC cells, but only those that are SMAD4 deficient^[53]^. Despite this, only a modest effect of olaparib in combination with radiation, versus radiation alone, was observed on growth of SMAD4-deficient HNSCC xenografts. Nevertheless, we have reported that two HPV-negative HNSCC cell lines were significantly radiosensitised with olaparib (0.1 µmol/L) with dose enhancement ratios (DER) of 1.79 and 3.34^[25]^. This was in comparison to two HPV-positive HNSCC cell lines, whereby only one of these showed increased radiosensitisation in the presence of olaparib (DER = 1.51). A separate study using five HPV-positive HNSCC cell lines displayed increased radiosensitivity after olaparib (1 µmol/L) treatment, although markedly only in four cell lines following a relatively high radiation dose (6 Gy)^[54]^, suggesting that the radiation dose plays a fundamental role together with the DNA repair capacity of the cells. Using the PARP inhibitor niraparib (1 µmol/L), this was recently shown to equally increase the sensitivity of two HPV-positive and two HPV-negative HNSCC cell lines to conventional radiation (DER = 1.06-1.21) and proton irradiation (DER = 1.12-1.31)^[55]^. Both sets of cell lines displayed higher numbers of residual 53BP1 foci present when niraparib was used in combination with either form of radiation. In contrast, another recent study has demonstrated that niraparib (1 µmol/L) had a greater impact of the radiosensitisation of two HPV-negative (DER = 1.94-2.13) compared to two HPV-positive (DER = 1.36-1.43) HNSCC cell lines^[56]^. Cumulatively, although positive results are observed when HNSCC cells are treated with PARP inhibitors prior to irradiation, further studies are required to investigate in depth the potential selectivity towards HPV-positive and HPV-negative HNSCC, and therefore provide evidence for PARP as a potentially promising therapeutic target in the clinical setting.

### DSB repair

Targeting the proteins involved in the repair of DSBs, that are potentially toxic lesions induced by RT, is considered a promising strategy in HNSCC treatment. As detailed previously, HPV-positive HNSCC in general have been found to be DSB repair deficient which contributes to their increased radiosensitivity *in vitro* and *in vivo*, and so strategies have mostly focused on enhancing radiosensitivity of relatively radioresistant HPV-negative HNSCC. Given that DSBs are sensed by the protein kinases ATM, ATR, and DNA-Pkcs, these enzymes have been investigated as targets for inhibitors to increase HNSCC radiosensitisation. A number of studies have focused on ATR and found this to be a successful approach for increasing radiosensitivity in HPV-negative HNSCC cell lines. In particular, the ATR inhibitor VE821 was shown to enhance the radiosensitivity of a single HPV-negative HNSCC cell line in both normoxia and hypoxia (2% and < 0.02% oxygen)^[57]^. Similarly using the same inhibitor, we have also recently observed radiosensitisation of two oropharyngeal HPV-negative HNSCC cell lines grown as colonies but also as 3D spheroids following both photons and (low-LET) protons^[17]^. However, our study also demonstrated that a hypopharyngeal HPV-negative HNSCC spheroid model was not radiosensitized in the presence of VE821, suggesting cell and tumour-specific radiosensitisation. Using the ATR inhibitor AZD6738, two studies have demonstrated that HPV-negative HNSCC cell lines (four in total) can be radiosensitised following ATR inhibition, including the impact on preventing 3D spheroid growth of one of the cell lines^[58,59]^. The combination of both ATR and DNA-Pkcs inhibition (KU-0060648) further exacerbated the cell killing effects post-irradiation^[59]^. In relation to ATM as a target for radiosensitisation in HNSCC cells, the evidence is sparser. A small molecule inhibitor screen identified the compound GSK635416A in enhancing the radiosensitivity of three HPV-negative HNSCC cell lines, which was then demonstrated to act through inhibition of ATM^[60]^. This study also showed evidence for additive effects of ATM inhibition in combination with PARP inhibitor (olaparib) in enhancing radiosensitivity. Data recently generated in our lab has shown a significant impact of the ATM inhibitor KU-55933 in reducing survival of four HPV-negative HNSCC cell lines (two derived from the oropharynx and one each from the hypopharynx and oral cavity) and reducing growth of these 3D spheroid models in response to both photons and protons^[17]^. Targeting ATM was also shown to enhance the radiosensitivity of one HPV-positive HNSCC cell line, but not a second which was the most radiosensitive cell line used.

Several studies have identified that DNA-Pkcs inhibition using different compounds has the ability to increase HNSCC radiosensitivity. The DNA-Pkcs inhibitors KU0060648^[59]^ and IC87361^[61]^ have both been shown to cause reduced survival of HPV-negative HNSCC cell lines (five in total) post-irradiation, the latter study also suggesting that this approach was more effective than the combination of radiation with targeting PARP (via olaparib). Evidence using the DNA-Pkcs inhibitor NU7441 has further demonstrated the effectiveness of targeting DNA-Pkcs in radiosensitising three HPV-negative in addition to three HPV-positive HNSCC cell lines^[27]^, suggesting that this is a more general strategy for treatment of HNSCC. This study was further progressed *in vivo*, where a significant delay in growth of a HPV-positive xenograft model was observed. The slow growth of a HPV-negative xenograft model did not allow for an accurate assessment of the effectiveness of the combination of the DNA-Pkcs inhibitor with radiation, although this was shown in two patient-derived xenograft models where tumour growth was significantly suppressed. Finally, our recent evidence has demonstrated that the DNA-Pkcs inhibitor KU-57788 significantly enhanced the radiosensitisation of four HPV-negative HNSCC cell lines, including suppression of the growth of 3D spheroid models, following both photons and protons^[17]^. We also observed increased radiosensitisation of HPV-positive HNSCC cells and spheroids, and in general identified that targeting DNA-Pkcs was the most effective strategy for radiosensitisation of HNSCC cells in comparison to inhibitors against ATM and ATR. Cumulatively, these data suggest that targeting the key proteins involved in the repair of DSBs, particularly ATR and DNA-Pkcs, may be an effective treatment in combination with RT for HPV-negative HNSCC. Nevertheless, further evidence investigating this strategy in a broader range of HNSCC cell lines *in vitro*, as well as utilising *in vivo* models, are necessary.

### Cell cycle

Checkpoint activation allows halting of cell cycle progression and is therefore important in responding to radiation-induced DNA damage to allow cells to accomplish DNA repair. Notably, due to disruption of p53/pRb and cell cycle regulation caused by HPV infection, the proteins involved in cell cycle checkpoint activation, particularly CHK1 and WEE1 in the G2/M checkpoint, are of therapeutic interest in causing enhanced RT treatment of HPV-positive HNSCC. However, this approach has also been investigated in pre-clinical studies in HPV-negative models of the disease. Initial evidence using the CHK1 inhibitor SAR020106 showed enhanced radiosensitivity of two HPV-negative HNSCC cell lines *in vitro* and tumour growth delay of a HPV-negative HNSCC model *in vivo*^[62]^. This is supported by a study using the CHK1 inhibitor CCT244747 in two HPV-negative HNSCC cell lines where increased radiosensitivity was observed, along with delayed growth of an HPV-negative HNSCC xenograft model^[63]^. However, this study also identified that the triple combination of paclitaxel, CHK1 inhibitor, and radiation was able to significantly reduce xenograft tumour growth. Subsequent studies using the CHK1 inhibitor PF0477736 demonstrated increased radiosensitisation in two out of three HPV-negative HNSCC cell lines associated with loss of distal chromosome 11q^[64]^, and only one out of two HPV-negative HNSCC cell lines^[65]^. The latter study in fact showed that four out of five HPV-positive HNSCC cell lines tested were radiosensitised with CHK1 inhibition, which was supported by a follow-up study performed by the same research group in two HPV-positive HNSCC cell lines^[54]^. Additional evidence acquired in three HPV-positive HNSCC cell lines showed the impact of the CHK1 inhibitors LY2603618 and MK8776 in increasing cellular radiosensitivity, but also that the additive effect of the WEE1 inhibitor AZD1775 was relatively mild^[66]^. Interestingly, the WEE1 inhibitor alone in combination with radiation only appeared effective in radiosensitising one of the three HPV-positive HNSCC cell lines.

Studies have also examined combinatorial strategies using inhibitors targeting both cell cycle checkpoints, and DNA repair (via PARP). Here, the additive effect of PARP inhibition (using olaparib) in combination with CHK1 inhibitor PF00477736 in reducing radiation-induced survival of two HPV-positive HNSCC cell lines has been shown^[54]^. This is supported by a more recent study demonstrating the added benefit of CHK1 (MK8776) and PARP (niraparib) inhibition in enhancing radiosensitivity of one HPV-positive HNSCC cell line, but which was not effective in one HPV-negative HNSCC cell line^[56]^. In contrast, it was additionally observed that the HPV-negative HNSCC cell line was more susceptible to the combination of PARP and WEE1 (MK1775) inhibition, which was suggested to be due to more potent activation of the G2/M checkpoint caused by WEE1 inhibition that is downstream of CHK1. Noteworthy, a phase I dose-finding multicenter trial has begun this year with the aim of determining a safe dose of the WEE1 inhibitor AZD1775, both for preoperative patients treated with cisplatin and for post-operative patients treated with cisplatin-based chemoradiation^[102]^.

Another promising therapeutic target for radiosensitising HNSCC is CDK4/6, utilising the inhibitors palbociclib and ribociclib. Palbociclib has been demonstrated to selectively increase the radiosensitisation of HPV-negative, but not HPV-positive HNSCC cell lines both in normoxic and in hypoxic (1%) conditions^[67]^. This treatment was shown to cause a reduction in HR efficiency post-irradiation, ultimately leading to increased chromosomal damage and cell death. Palbociclib is currently under testing in the UPSTREAM trial in patients with recurrent or metastatic HNSCC who show cyclin D1 amplification and p16 negativity. Ribociclib is a small inhibitor of CDK4/6, which has showed promising safety and efficacy results in a phase I study in patients with local or metastatic recurrence of HNSCC together with cetuximab^[103]^. However, the impact of ribociclib in combination with radiation on HNSCC cell models has yet to be investigated. Given the promising evidence that targeting the cell cycle can enhance the radiosensitivity of HNSCC cell lines, particularly relatively radioresistant HPV-negative HNSCC, more comprehensive studies are warranted to accumulate this important preclinical data.

## Conclusion

Promising data has been reported demonstrating that the radiosensitivity of HNSCC, particularlyrelatively radioresistant HPV-negative HNSCC, can be increased by inhibitors targeting proteins involved in DNA damage repair (PARP, ATR, and DNA-Pkcs) and in DNA damage checkpoint activation (CHK1). These preclinical studies have largely been performed *in vitro* using the large numbers of established HNSCC cell lines that exist^[104]^, but that variability in the degree of radiosensitisation is observed which is dependent on the specific cell line used. These studies also differ in the precise inhibitors utilised that harbour different selectivity and potency. Therefore, the challenge now is to accumulate preclinical evidence using the most selective and up to date inhibitors in a larger cohort of HNSCC models, to convincingly demonstrate that such approaches can enhance the radiosensitivity of HNSCC. These models should include HPV-positive and HPV-negative cell lines from the oropharynx, but also those originating from different tumour origins, including the larynx, hypopharynx, and oral cavity which are largely HPV-negative, to understand whether or not the strategies investigated can be utilised more generally in treating HNSCC. It is also apparent that more advanced HNSCC models should be employed in this research, including 3D spheroids and well characterized patient-derived organoids, that mimic more precisely the structure and environment of the original HNSCC from which they were obtained. Whilst 3D spheroids can be developed from the current HNSCC cell lines that exist [Table 1], these should be utilised more routinely in research. However, there is a current lack of availability of patient-derived HNSCC organoids although some have recently been reported^[105,106]^. Additionally, radiosensitisation strategies should be investigated in *in vivo* models, particularly xenografts of HNSCC, to provide clear evidence that targeted inhibitors are effective at suppressing or preventing HNSCC tumour growth. Another interesting aspect to examine is the impact of particle beam therapy (e.g., protons and carbon ions) both alone and in combination with targeted inhibitors. As we detailed earlier, these data are currently sparse, mainly due to limited access to the appropriate resources, although there is some evidence to suggest that protons in particular may have a different impact on the DNA damage spectra induced compared to conventional photon irradiation, and thus may respond differently to combinations with DNA repair and DNA damage checkpoint inhibitors. Similarly, the impact of increasing LET at and around the Bragg peak and the effect of higher-LET radiation, including carbon ions, has yet to be thoroughly investigated. Nevertheless, once this extensive preclinical data has been acquired for clearly identifying effective strategies in combination with RT (photons and particle ions) for HNSCC, this will pave the way forward for future Phase I/II clinical trials.

## Declarations

### Copyright

© The Author(s) 2020.
